# Assessment of exposure to *Plasmodium falciparum *transmission in a low endemicity area by using multiplex fluorescent microsphere-based serological assays

**DOI:** 10.1186/1756-3305-4-212

**Published:** 2011-11-07

**Authors:** Jean Biram Sarr, Eve Orlandi-Pradines, Sonia Fortin, Cheikh Sow, Sylvie Cornelie, François Rogerie, Soihibou Guindo, Lassana Konate, Thierry Fusaï, Gilles Riveau, Christophe Rogier, Franck Remoue

**Affiliations:** 1 MIVEGEC (UM1-CNRS 5290-IRD 224), Montpellier, France; 2 ONG Espoir Pour La Santé (EPLS), Saint-Louis, Senegal; 3 Laboratoire d'Ecologie Vectorielle et Parasitaire (LEVP), UCAD, Dakar, Senegal; 4 IRBA & UMR6236, Marseille, France; 5 Institut Pasteur de Madagascar, Antananarivo, Madagascar

## Abstract

**Background:**

The evaluation of malaria transmission intensity is a crucial indicator for estimating the burden of malarial disease. In this respect, entomological and parasitological methods present limitations, especially in low transmission areas. The present study used a sensitive multiplex assay to assess the exposure to *Plasmodium falciparum *infection in children living in an area of low endemicity. In three Senegalese villages, specific antibody (IgG) responses to 13 pre-erythrocytic *P. falciparum *peptides derived from Lsa1, Lsa3, Glurp, Salsa, Trap, Starp, Csp and Pf11.1 proteins were simultaneously evaluated before (June), at the peak (September) and after (December) the period of malaria transmission, in children aged from 1 to 8 years.

**Results:**

Compared to other antigens, a high percentage of seropositivity and specific antibody levels were detected with Glurp, Salsa1, Lsa3NR2, and Lsa1J antigens. The seropositivity increased with age for all tested antigens. Specific IgG levels to Glurp, Salsa1, Lsa3NR2, and Lsa1J were significantly higher in *P. falciparum *infected children compared to non-infected and this increase is significantly correlated with parasite density.

**Conclusion:**

The multiplex assay represents a useful technology for a serological assessment of rapid variations in malaria transmission intensity, especially in a context of low parasite rates. The use of such combined serological markers (i.e. Glurp, Lsa1, Lsa3, and Salsa) could offer the opportunity to examine these variations over time, and to evaluate the efficacy of integrated malaria control strategies.

## Background

*Plasmodium falciparum *malaria is a major cause of human morbidity and mortality in sub-Saharan Africa, and its transmission varies greatly in endemicity across the continent [1]. The expanding utilization of combined malaria control strategies including insecticide impregnated bednets and artemisinin combination therapies, has contributed to greatly reduce malaria transmission in several sub-Saharan African areas [2,3]. Consequently, the current methods for evaluating malaria transmission intensity (MTI), such as entomological inoculation rate and *Plasmodium *parasitemia in human populations, present substantial limitations, e.g. reproducibility and can be time-consuming. In addition, both entomological and parasitological measures are affected by the seasonality and require a precise follow-up during longitudinal studies [4]. For this purpose, there is an increased need for developing new tools for the monitoring of MTI in more frequent contexts of low malaria transmission. In this respect, the sero-epidemiological approach offers a theoretical advantage over parasite prevalence for assessing MTI or changes in prevalence following the implementation of control programmes [5]. In order to identify *Plasmodium *infections, serological markers show greater sensitivity, as seroprevalence reflects cumulative exposure to infections and thus is less affected by the changes in parasite densities, which could be undetectable in the case of very low parasite density. Previous studies showed that serological measurements are robust to detect short term variations in transmission, and should be a pertinent tool for evaluating malaria exposure in the context of low transmission [6].

The Circumsporozoite protein (CSP: a protein expressed by sporozoites and early liver forms), has been frequently used for the serological estimation of MTI [7]. Controversial studies have reported that antibody (Ab) responses directed to the repetitive NANP domains of CSP remained very low throughout the first year of life [8]. In addition, it has been suggested that human immunological memory following malaria infection is short-lived because Ab responses rapidly decline after the end of the transmission season or exposure period [9], and after treatment of a clinical episode [10]. It suggests that maintenance of immunological memory therefore requires antigen persistence and may be age-dependent [11]. For this purpose, it has been then demonstrated that the simultaneous use of several antigens (Ags) as serological markers could lead to a better evaluation of malaria exposure than using only one Ag, i.e. CSP [12]. Human Ab levels to plasmodial Ags are classically assessed using the enzyme-linked immunosorbant assay (ELISA) test. This technique is labour-intensive and time-consuming, as well as requiring considerable quantity of Ags and sera samples. An immunoassay that measures Ab to multiple Ags simultaneously would be highly advantageous [13]. Multiplexed bead assays gives similar sensibility than ELISA assays, and have been developed in several studies for simultaneous detection of Ab against multiple plasmodial Ags in humans living in endemic areas [14,15].

In the present study, we used multiplex fluorescent microsphere-based assays measuring simultaneously human Ab to thirteen *P. falciparum *peptides [14] to assess malaria transmission in children living in a low endemicity area. All Ags used in the assay have previously been shown to be antigenic and associated with malaria transmission in individuals living in malaria endemic areas [6,14].

## Materials and methods

### Study population

The study was performed in the villages of Mboula (Ferlo area: 15°40' N, 15°25' W), Gankette Balla (near the Guiers Lake: 15°58' N, 15°55' W) and Mbilor (Low valley area: 16°29' N, 15°33' W) in Northern Senegal, located along the Senegal Basin River [16]. This site is a dry savannah area, with approximately 400 mm of rain by year. Malaria transmission is low (average of 3 to 7 infective bites per human per year), markedly seasonal (occurring from July to October), with a peak in September [16].

Three cross-sectional surveys were performed in June, September and December 2004 in the three villages. For each passage, a sub-cohort of children aged from 1 to 8 years was randomly selected from an existing cohort of 450 children, as previously described [17]. A total of 186 children (Mboula n = 64; Gankette n = 56 and Mbilor n = 66, selected for the 3 passages) were included in the present study. For each child, parasitological measurements of malaria were performed at each passage using thick blood smears obtained by finger-prick. The smears were Giemsa stained to identify *Plasmodium *species and the number of malaria parasites was counted in 200 microscopic fields. Parasite density was defined as the number of *P. falciparum *parasites/μl of blood, corresponding to an average of 8000 leucocytes. In the same way, capillary blood was collected from each child at each passage for immunological assessments.

The present study followed ethical principles according to the Helsinki Declaration, and was approved by the Ethics Committee of the Ministry of Health of Senegal (CNRS, June 2004). Informed consent was obtained from the parents/tutors of the children.

### Multiplex bead-based assay

The multiplex technique was performed using the same sequences of *P. falciparum *peptides (Lsa1-41, Lsa1J, Lsa3NR2, Lsa3RE, Glurp, Glurp.P3, Salsa1, Salsa2, Trap1, Trap2, StarpR, Csp, Sr11.1), and the procedure for coupling Ags to beads, as previously described [14]. Briefly, the *P. falciparum *peptides were synthesized with an added N-terminal cysteine residue and covalently coupled with bovine serum albumin by Genepep (Ales, France). These Ags were coupled to beads (Biorad Inc, CA, USA) and 50 μl/well was deposited at the final concentration of 80 beads/μl per peptide. Diluted individual sera (1/100) in equal volumes of PBS and MFIA (Multiplex Fluorescence ImmunoAssay) diluents (Charles River Laboratories Inc, MA, USA) were added in duplicate using 50 μl/well. The Luminex system was set for reading a minimum of 100 beads per spectral address, and results were expressed as ΔMFI "median fluorescent intensity" value with ΔMFI = MFI_Ag _- MFI_BSA_, where MFI_Ag _represents the mean of individual MFI value for beads coupled with *P. falciparum *Ag, and MFI_BSA _the individual MFI value for each serum for beads without coupled Ag. For calculating the seropositivity threshold, the means and standard deviations (SD) of ΔMFI of individuals living permanently in a non-endemic area (*i.e*. negative control group - n = 19) were estimated for each Ag. The lower limit of positivity of Ab responses to one Ag was estimated as the mean of ΔMFI of the negative control group + 3.09 SD. Therefore, values higher than the cut-offs should be observed in less than 1% of sera of non-exposed individuals under the hypothesis of normally distributed of ΔMFI. The thresholds of positivity were 7466.6, 684.0, 725.0, 225.0, 344.2, 4593.1, 424.8, 439.7, 435.2, 933.6, 452.6, 380.2, and 302.5 respectively for Lsa1-41, Lsa1J, Lsa3NR2, Lsa3RE, Glurp, Glurp.P3, Salsa1, Salsa2, Trap1, Trap2, StarpR, Csp, and SR11.1. Individual time-period variations in Ab levels for each Ag were assessed by the ratio of ΔMFI values collected at different time intervals, i.e. between June - September; September - December or June - September. The significant increase in Ab level to each Ag was defined as a value superior to the mean + 3.09 SD of the ratio in ΔMFI in the negative control group collected two times at four months interval.

### Statistical analysis

All data were analyzed with Graph Pad Prism^® ^(Graph Pad, San Diego, USA) version 4. After checking for normal distribution, the proportions of Ab-positive individuals and Ab levels were analyzed using the chi-squared test, Mann-Whitney U-test, Kruskal-Wallis test, where appropriate. Spearman's correlation was used to check for correlations between continuous variables. Differences were considered significant at P < 0.05.

## Results and discussion

**Figure 1:**
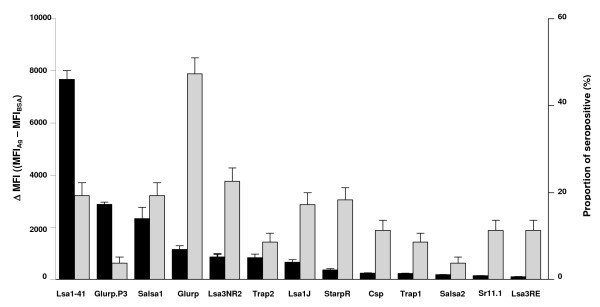
**Proportion of seropositive and Ab levels to *P. falciparum *peptides (n = 186)**. Black and gray bars indicate respectively the Ab levels (ΔMFI) and proportion of seropositive. Error bars indicate the upper limits of the 95% confidence intervals.

The proportion of seropositive and the level of specific Ab (IgG) responses directed to different *P. falciparum *peptides are presented for all studied individuals (Figure 1). A high IgG level was found in the majority of peptides tested. Lsa1-41 presented the highest Ab responses, i.e. antigenicity, with a ΔMFI mean of 7673 (95% CI = 7010 - 8336). Some other pre-erythrocytic (GlurpP3, Salsa1, Glurp, Lsa3NR2, Trap2 and Lsa1J) peptides were associated with moderate Ab response levels with a ΔMFI values ranging from 665 (95% CI = 473.9 - 857.1) to 3000 (95% CI = 2890 - 3265). In contrast, StarpR, Csp, Trap1, Salsa2, Sr11.1 and Lsa3RE peptides presented lower levels of antigenicity. The proportion of seropositive was markedly higher for the Glurp peptide. Moderate seroprevalence was observed for Salsa1, Lsa1-41, Starp, Lsa3NR2, and Lsa1J peptides, whereas a very low proportion of seropositive was observed for all other Ags. These results suggested that pre-erythrocytic Lsa1-41, Salsa1, Glurp, Lsa1J and Lsa3NR2 peptides appeared to be most antigenic, giving the highest proportion of seropositive, and therefore could be potential serological markers of malaria transmission. However, it can not be excluded that differences in antigenicity levels (ΔMFI values) between these peptides could be due also to the different structure of repetitive epitopes and to their conformation when they are coupled to the beads, as previously indicated [18]. Furthermore, the specific Ab response levels and the seropositivity (Table 1) to these relevant antigens (Lsa1-41, Salsa1, Glurp, Lsa1J and Lsa3NR2) were investigated according to the village and period of malaria transmission. No statistical influence of village and of time-period on specific Ab level (data not shown) and seropositivy were observed. These results could be attributed to the similar prevalence of *P. falciparum *in children in June (37.3%, n = 22), September (40.3%, n = 25) and December (43.1%, n = 28) and according to the village (Mboula 45.3%, n = 29; Gankette 41.1%, n = 23; Mbilor 34.9%, n = 23).

**Table 1:** Proportion and number of seropositive according to the period of malaria transmission and village.

	Mboula (n = 64)			Gankette (n = 56)			Mbilor (n = 66)			All villages (n = 186)		
	June	Sept	Dec	June	Sept	Dec	June	Sept	Dec	June	Sept	Dec
**Lsa1-41**	6.7% (1/15)	18.2% (4/22)	25.9% (7/27)	22.2% (4/18)	26.3% (5/19)	36.8% (7/19)	7.7% (2/26)	14.3% (3/21)	15.8% (3/19)	11.9% (7/59)	19.3% (12/62)	26.1% (17/65)
**Salsa1**	26.7% (4/15)	9.1% (2/22)	22.2% (6/27)	22.2% (4/18)	15.8% (3/19)	15.8% (3/19)	23.1% (6/26)	23.8% (5/21)	15.8% (3/19)	23.7% (14/59)	16.1% (10/62)	18.5% (12/65)
**Glurp**	46.7% (7/15)	31.8% (7/22)	48.1% (13/27)	44.4% (8/18)	42.1% (8/19)	31.6% (6/19)	61.5% (16/26)	47.6% (10/21)	68.4% (13/19)	52.5% (31/59)	40.3% (25/62)	49.2% (32/65)
**Lsa1J**	33.3% (5/15)	31.8% (7/22)	29.6% (8/27)	16.7% (3/18)	15.8% (3/19)	21.0% (4/19)	7.7% (2/26)	4.8% (1/21)	5.3% (1/19)	16.9% (10/59)	17.7% (11/62)	20.0% (13/65)
**Lsa3NR2**	6.7% (1/15)	9.1% (2/22)	25.9% (7/27)	27.8% (5/18)	31.6% (6/19)	36.8% (7/19)	11.5% (3/26)	38.1% (8/21)	10.5% (2/19)	15.2% (9/59)	25.8% (16/62)	24.6% (16/65)

The prevalence rate of positive *P. falciparum *infection was 37.3%, 40.3%, 43.1% respectively at June, September, December and 45.3%, 41.1% and 34.9% respectively in Mboula, Gankette and Mbilor villages.

**Figure 2:**
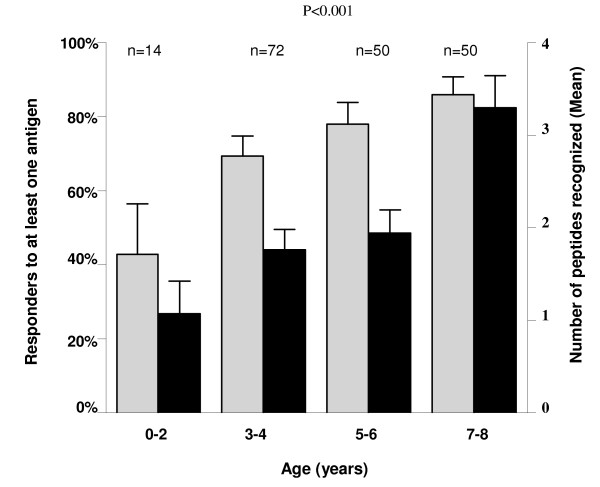
**Number of *P. falciparum *peptides that produced seropositive results and proportion of seropositive to at least one peptide, according to the age**. Gray and black bars indicate respectively the proportion of responders to at least one peptide and the mean number of peptides that showed seropositive results. Error bars indicate the upper limits of the 95% confidence intervals. *P *value of the Kruskal-Wallis test.

In the objective to explore the possibility that these potential serological markers could reflect exposure to malaria over a prolonged period, the proportion of seropositive to at least one peptide, and the number of peptides that showed seropositivity in children were analyzed according to age (Figure 2). The seropositivity to at least one peptide increased significantly according to age group, and remained higher in older (> 5 years) than in younger (≤5 years) children (P < 0.001). The number of peptides for which children developed high IgG titers was significantly age-dependant (P < 0.001). Lsa1-41, Salsa1, Glurp, Lsa3NR2, Trap2, Csp, Trap1, Salsa2 and StarpR peptides showed a significant increase of seropositivity with age (all P < 0.02). In addition, in older children (7-8 years) who were seropositive to at least one peptide, 93.0% (40 of 43) were seropositive to at least one of 4 specific peptides: Lsa1-41, Salsa1, Glurp and Lsa3NR2. These results suggested that seropositivy to peptide reflects cumulative exposure to malaria infection, and that it could need more than one peptide for assessing MTI. The need to use several peptides for pertinent MTI evaluation appeared relevant especially in young children (< 5 years), as previously described [19].

**Figure 3:**
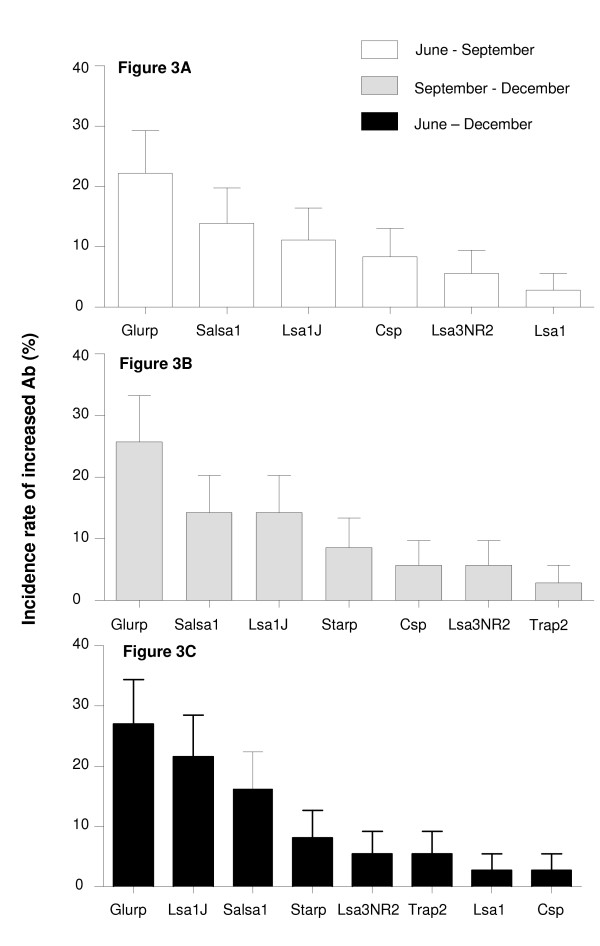
**Incidence of significant increases in specific Ab to *P. falciparum *peptides (Glurp, Salsa1, Lsa1J, Starp, Csp, Lsa3NR2, Lsa1, and Trap2) between June - September (Figure 3A, n = 33); September - December (Figure 3B, n = 32) and June - December 2004 (Figure 3C, n = 33)**. For all other peptides, no significant increase in Ab was observed during the period of malaria transmission. Error bars indicate the upper limits of the 95% confidence intervals.

The incidence rate of a significant increase in IgG level to *P. falciparum *peptides is shown according to the 3 time-periods, i.e. between June - September (Figure 3A); September - December (Figure 3B) and June - December 2004 (Figure 3C). Significant increase of specific IgG levels to at least one peptide was observed for 33.3% (95% CI = 17.2-49.5%), 45.7% (95% CI = 28.3-63.1%) and 51.3% (95% CI = 34.4-68.2%) of individuals, between June-September; September-December and June-December, respectively. In particular, IgG levels to Glurp, Lsa1J, Salsa1, Csp, and Lsa3NR2 increased significantly whatever the time-period studied. In contrast, no increase in Ab was observed for Lsa3RE, Salsa2, Sr11.1, Glurp.P3 and Trap1 (data not shown). Among the 47 individuals presenting a significant increase of Ab to at least one peptide between the different periods, 25 (53.2%), 16 (34.0%), 15 (31.9%), 6 (12.8%), 6 (12.8%), 5 (10.6%) showed an increased IgG to Glurp, Lsa1J, Salsa1, Starp, Lsa3NR2 and Csp respectively. Moreover, the incidence rate of increased IgG levels to Glurp, Salsa1 and Lsa1J, were significantly higher compared to others peptides (all P < 0.02), and then, whatever the periods of malaria transmission considered. Altogether, these results suggested that Glurp, Salsa1, Lsa1J, Lsa3NR2 and Csp peptides could be the relevant candidates for evaluating potential season-dependent variations of malaria exposure. In addition, the increase of the number of Ags tested may provide a better estimation of malaria transmission, particularly in areas of low endemicity.

**Figure 4:**
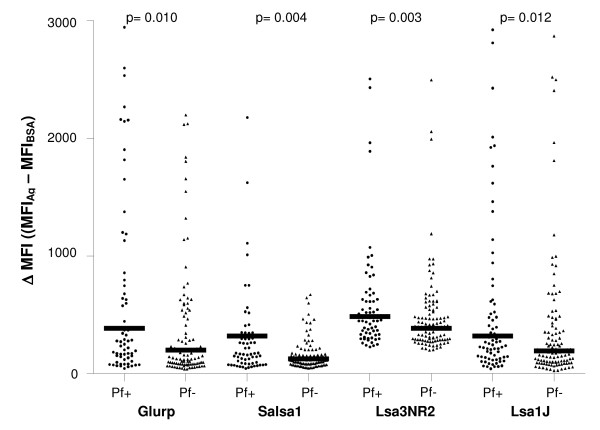
**Differences in specific Ab response directed to Glurp, Salsa1, Lsa3NR2 and Lsa1J peptides according to the presence (n = 75) or absence (n = 111) of *P. falciparum *infection**. Pf + and Pf - = *P. falciparum *positive and negative children. *P *value of the Mann-Whitney U-test.

Specific Ab levels to *P. falciparum *peptides were compared according to the presence of malaria infection in children (Figure 4). Positive children for *P. falciparum *(n = 75), presented higher specific Ab response to Glurp, Salsa1, Lsa3NR2, and Lsa1J peptides (all *P *< 0.05) than negative children (n = 111). In *P. falciparum *infected children, 80.0%, 54.7%, 62.7%, and 50.7% were seropositive to Glurp, Lsa3NR2, Salsa1 and Lsa1J respectively. Moreover, 85.3% of *P. falciparum *infected children were seropositive for at least one of the candidate antigens (Glurp, Lsa3NR2, Salsa1, and Lsa1J). For other peptides, no significant differences of Ab response levels were observed between *P. falciparum *positive and negative children (data not shown). Moreover, the highest differences in specific IgG levels were respectively observed for Glurp, Salsa1, Lsa3NR2 and Lsa1J. The statistical correlation between specific IgG level and the *P. falciparum *parasite density indicated a positive and significant correlation for Glurp, Salsa1, Lsa3NR2, and Lsa1J (all *r *> 0.320, all *P *< 0.01). Altogether, these data suggested that Glurp, Salsa1, Lsa3NR2 and Lsa1J peptides seem to be closely associated with *P. falciparum *parasite presence and intensity, and could be therefore potential serological markers of malaria exposure. This positive association between *P. falciparum *infection and specific Ab response against plasmodial antigens is a consistent observation, and could result from a booster effect of sustained parasite-exposure [6,20-22].

Some of these antigens (Glurp, Lsa1J, Lsa3NR2, and Salsa1) appeared to satisfy several of the requirements expected for a relevant marker of MTI. These peptides are antigenic and provided a higher incidence rate of significant increases in IgG levels. Specific Ab levels to these Ags are closely associated with the presence and the intensity of malaria infection. Moreover, in children seropositive to at least one peptide, 86.3% (120 of 139) were seropositive to at least one out of these four peptides. Pre-erythrocytic Glurp, Lsa1, Lsa3 and Salsa Ags are mainly expressed in infected human hepatocytes, they present little polymorphism from geographically different *P. falciparum *isolates [23-26]. Recent immuno-epidemiological studies in different endemic areas, have shown a positive association of specific Ab directed to these Ags and the level of malaria transmission [14]. Our results strengthen that pre-erythrocyte Ags, and particularly Glurp, Lsa1, Lsa3, and Salsa, could be pertinent markers to assess and to survey malaria transmission, at the individual level and in the context of low *P. falciparum *prevalence. Nevertheless, further studies will be valuable for validating these potential serological markers. These studies could include future modelling exercises including covariates factors such as age, genetic background, village of residence, impact of vector/parasite control programmes and the period of transmission. This kind of tool could allow elaborating a precise picture of malaria epidemiology in large-scale areas, or be used as a complementary indicator for evaluating the efficacy of integrated malaria control strategies.

## Conclusion

In conclusion, the multiplex assay provides a useful tool to simultaneously measured Ab responses directed to several Ags used as potential markers of malaria transmission. In low transmission areas, serological measurements to various malaria antigens are needed for estimating short term and small scale variations in MTI. However, the present study suggests that the combined assessment of Ab levels to only Glurp, Lsa1, Lsa3, and Salsa Ags could be a pertinent serological marker for evaluating the MTI.

## Competing interests

The authors declare that they have no competing interests.

## Authors' contributions

All authors read and approved the final manuscript.

JBS carried out the immunological assessments, statistical analysis, interpretation of data and drafted the manuscript. CS, SF, SG contributed to field activities, microscopic examinations, and initial statistical analysis. SC helped to draft the manuscript. EO-P, FR1, LK, TF and CR contributed to the design of the study, and choice of the peptides. GR, CR, FR2 provided the scientific supervision, contributed to the design of the methods, interpretation of the data, and revised the manuscript.
